# Supplementary material for: Loss of Runx1 Induces Granulosa Cell Defects and Development of Ovarian Tumors in the Mouse
Source: Int J Mol Sci. 2022 Nov 21;23(22):14442. doi: 10.3390/ijms232214442 (PMC9697285; doi:10.3390/ijms232214442)
Supplement: Supplementary file 1 [file ijms-23-14442-s001.zip › ijms-1948124-supplementary figures.pptx]

## Slide 1
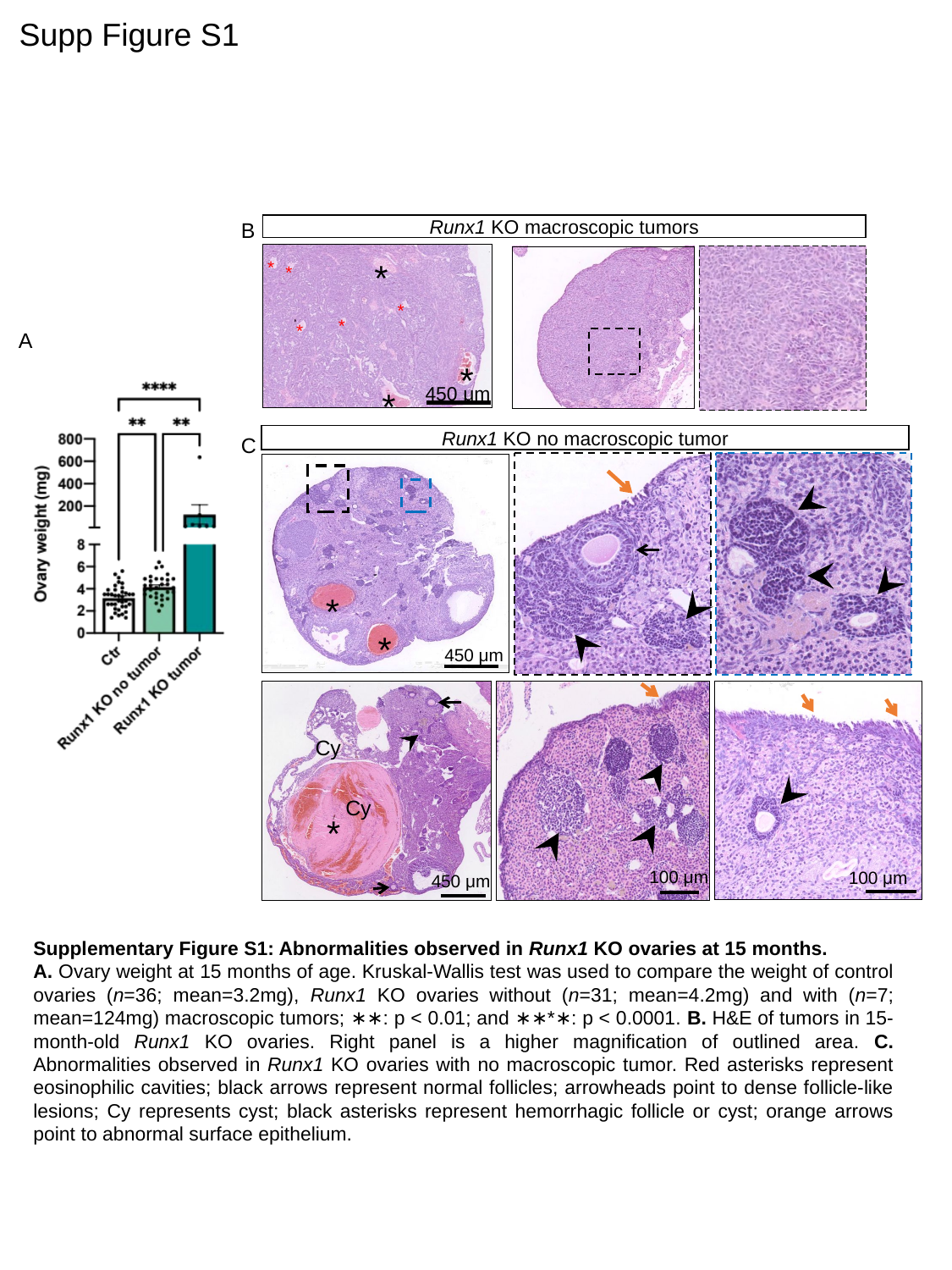

Supp Figure S1
B
Runx1 KO macroscopic tumors
450 μm
*
*
*
*
*
*
A
*
*
Runx1 KO no macroscopic tumor
C
*
*
450 μm
Cy
Cy
*
100 μm
100 μm
450 μm
Supplementary Figure S1: Abnormalities observed in Runx1 KO ovaries at 15 months.
A. Ovary weight at 15 months of age. Kruskal-Wallis test was used to compare the weight of control ovaries (n=36; mean=3.2mg), Runx1 KO ovaries without (n=31; mean=4.2mg) and with (n=7; mean=124mg) macroscopic tumors; ∗∗: p < 0.01; and ∗∗*∗: p < 0.0001. B. H&E of tumors in 15-month-old Runx1 KO ovaries. Right panel is a higher magnification of outlined area. C. Abnormalities observed in Runx1 KO ovaries with no macroscopic tumor. Red asterisks represent eosinophilic cavities; black arrows represent normal follicles; arrowheads point to dense follicle-like lesions; Cy represents cyst; black asterisks represent hemorrhagic follicle or cyst; orange arrows point to abnormal surface epithelium.

## Slide 2
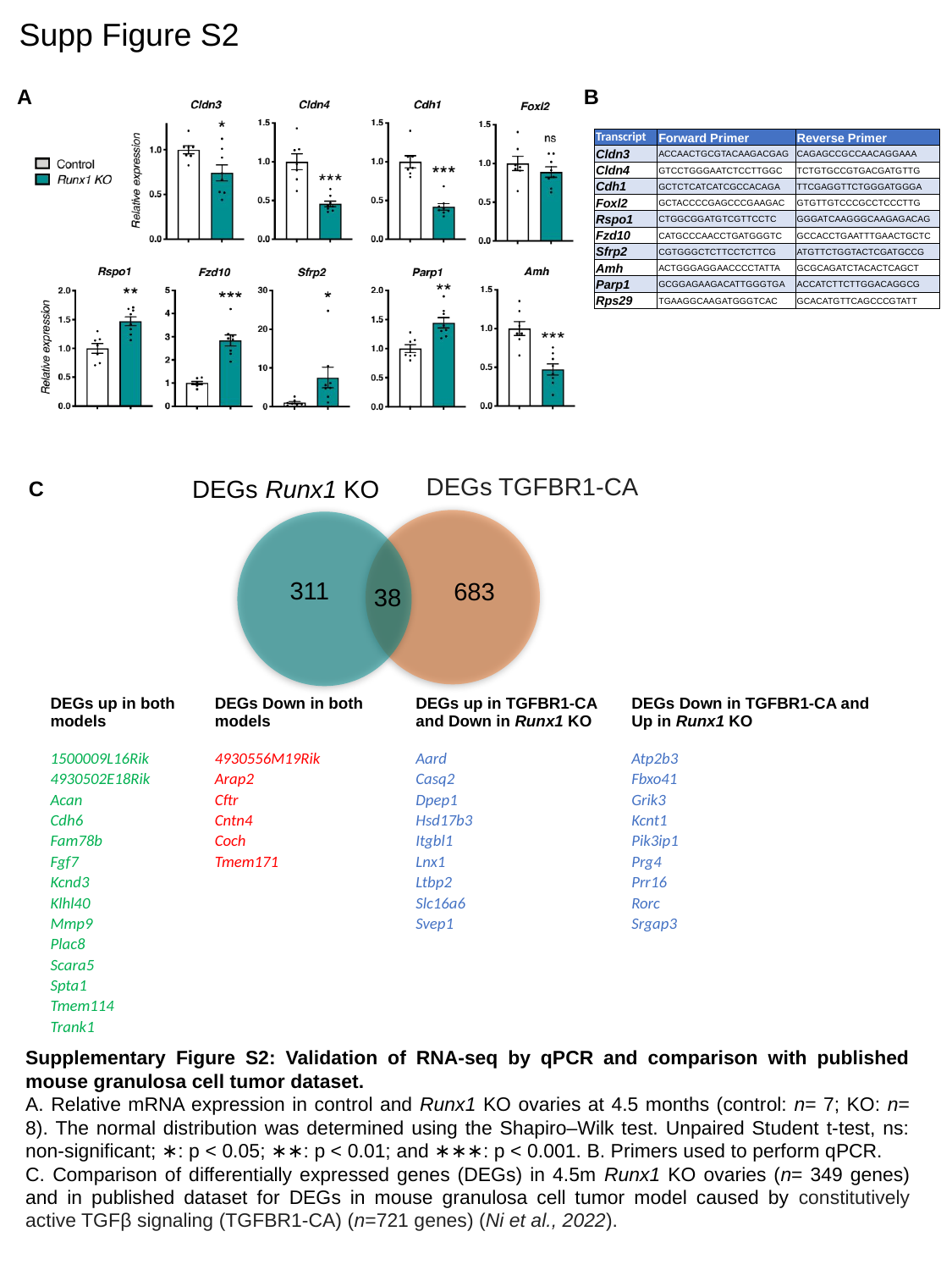

Supp Figure S2
B
A
| Transcript | Forward Primer | Reverse Primer |
| --- | --- | --- |
| Cldn3 | ACCAACTGCGTACAAGACGAG | CAGAGCCGCCAACAGGAAA |
| Cldn4 | GTCCTGGGAATCTCCTTGGC | TCTGTGCCGTGACGATGTTG |
| Cdh1 | GCTCTCATCATCGCCACAGA | TTCGAGGTTCTGGGATGGGA |
| Foxl2 | GCTACCCCGAGCCCGAAGAC | GTGTTGTCCCGCCTCCCTTG |
| Rspo1 | CTGGCGGATGTCGTTCCTC | GGGATCAAGGGCAAGAGACAG |
| Fzd10 | CATGCCCAACCTGATGGGTC | GCCACCTGAATTTGAACTGCTC |
| Sfrp2 | CGTGGGCTCTTCCTCTTCG | ATGTTCTGGTACTCGATGCCG |
| Amh | ACTGGGAGGAACCCCTATTA | GCGCAGATCTACACTCAGCT |
| Parp1 | GCGGAGAAGACATTGGGTGA | ACCATCTTCTTGGACAGGCG |
| Rps29 | TGAAGGCAAGATGGGTCAC | GCACATGTTCAGCCCGTATT |
DEGs TGFBR1-CA
DEGs Runx1 KO
311
683
38
C
| DEGs up in both models | DEGs Down in both models | DEGs up in TGFBR1-CA and Down in Runx1 KO | DEGs Down in TGFBR1-CA and Up in Runx1 KO |
| --- | --- | --- | --- |
| 1500009L16Rik | 4930556M19Rik | Aard | Atp2b3 |
| 4930502E18Rik | Arap2 | Casq2 | Fbxo41 |
| Acan | Cftr | Dpep1 | Grik3 |
| Cdh6 | Cntn4 | Hsd17b3 | Kcnt1 |
| Fam78b | Coch | Itgbl1 | Pik3ip1 |
| Fgf7 | Tmem171 | Lnx1 | Prg4 |
| Kcnd3 | | Ltbp2 | Prr16 |
| Klhl40 | | Slc16a6 | Rorc |
| Mmp9 | | Svep1 | Srgap3 |
| Plac8 | | | |
| Scara5 | | | |
| Spta1 | | | |
| Tmem114 | | | |
| Trank1 | | | |
Supplementary Figure S2: Validation of RNA-seq by qPCR and comparison with published mouse granulosa cell tumor dataset.
A. Relative mRNA expression in control and Runx1 KO ovaries at 4.5 months (control: n= 7; KO: n= 8). The normal distribution was determined using the Shapiro–Wilk test. Unpaired Student t-test, ns: non-significant; ∗: p < 0.05; ∗∗: p < 0.01; and ∗∗∗: p < 0.001. B. Primers used to perform qPCR.
C. Comparison of differentially expressed genes (DEGs) in 4.5m Runx1 KO ovaries (n= 349 genes) and in published dataset for DEGs in mouse granulosa cell tumor model caused by constitutively active TGFβ signaling (TGFBR1-CA) (n=721 genes) (Ni et al., 2022).

## Slide 3
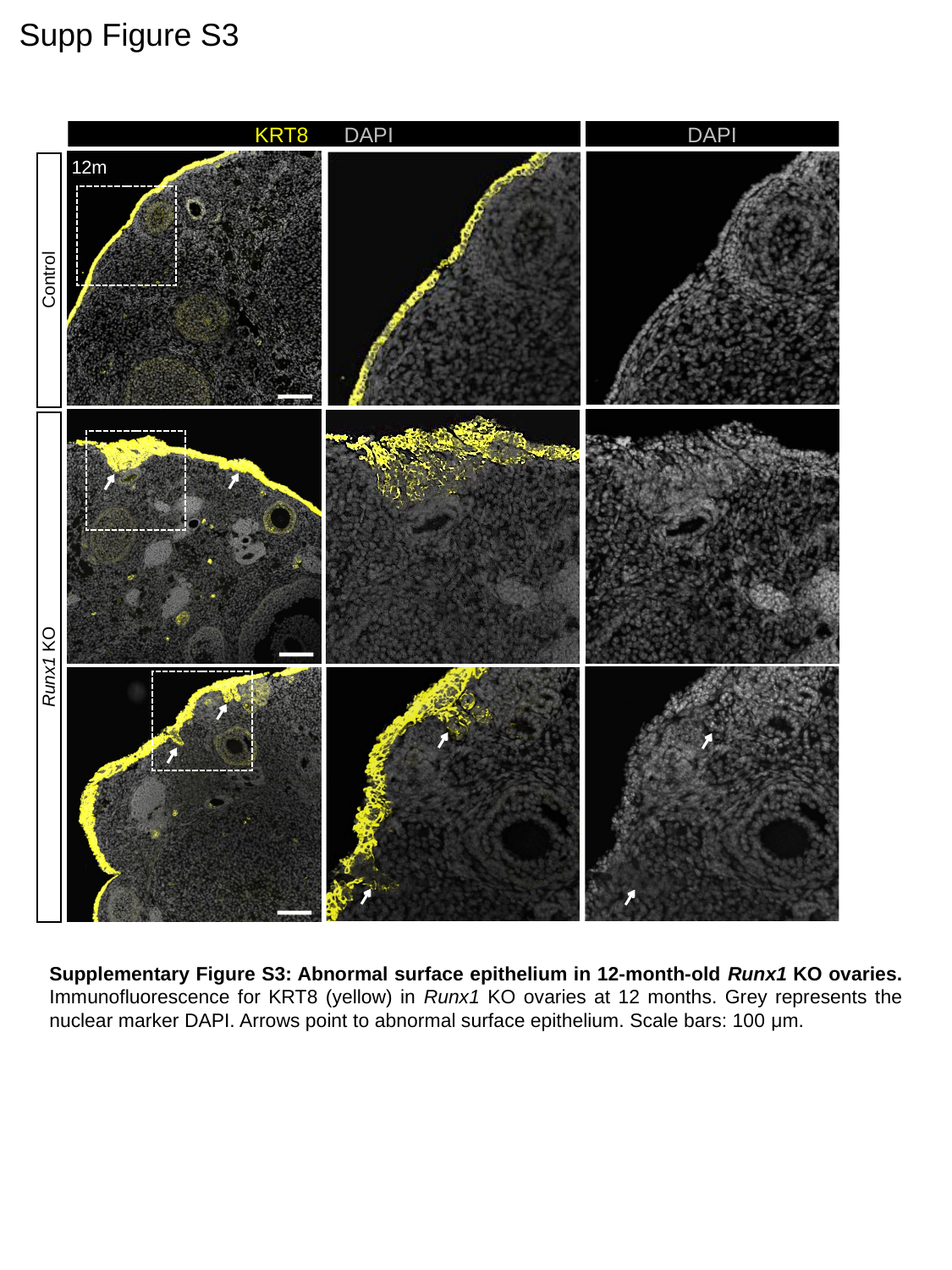

Supp Figure S3
KRT8 DAPI
DAPI
12m
Control
Runx1 KO
Supplementary Figure S3: Abnormal surface epithelium in 12-month-old Runx1 KO ovaries. Immunofluorescence for KRT8 (yellow) in Runx1 KO ovaries at 12 months. Grey represents the nuclear marker DAPI. Arrows point to abnormal surface epithelium. Scale bars: 100 μm.
